# All-trans retinoic acid impairs the vasculogenic mimicry formation ability of U87 stem-like cells through promoting differentiation

**DOI:** 10.3892/mmr.2015.3449

**Published:** 2015-03-06

**Authors:** GENG-QIANG LING, YI-JING LIU, YI-QUAN KE, LEI CHEN, XIAO-DAN JIANG, CHUAN-LU JIANG, WEI YE

**Affiliations:** 1 Department of Neurosurgery, Second Affiliated Hospital of Harbin Medical University, Harbin, Heilongjiang 150086, P.R. China; 2 Department of Neurosurgery, Zhujiang Hospital, National Key Clinic Department, Neurosurgery Institute, Key Laboratory on Brain Function Repair and Regeneration of Guangdong, Southern Medical University, Guangzhou, Guangdong 510282, P.R. China; 3 Department of Neurosurgery, The First Hospital of Putian, Teaching Hospital, Fujian Medical University, Putian, Fujian 351100, P.R. China; 4 Department of Neurosurgery, Shenzhen Second People’s Hospital, The First Affiliated Hospital of Shenzhen University, Shenzhen, Guangdong 518021, P.R. China

**Keywords:** all-trans retinoic acid, glioblastoma multiforme, vasculogenic mimicry, stem-like cells, vascular endothelial growth factor

## Abstract

The poor therapeutic effect of traditional antiangiogenic therapy on glioblastoma multiforme (GBM) may be attributed to vasculogenic mimicry (VM), which was previously reported to be promoted by cancer stem-like cells (SLCs). All-trans retinoic acid (ATRA), a potent reagent which drives differentiation, was reported to be able to eradicate cancer SLCs in certain malignancies. The aim of the present study was to investigate the effects of ATRA on the VM formation ability of U87 glioblastoma SLCs. The expression of cancer SLC markers CD133 and nestin was detected using immunocytochemistry in order to identify U87 SLCs. In addition, the differentiation of these SLCs was observed through detecting the expression of glial fibrillary acidic protein (GFAP), β-tubulin III and galactosylceramidase (Galc) using immunofluorescent staining. The results showed that the expression levels of GFAP, β-tubulin III and Galc were upregulated following treatment with ATRA in a dose-dependent manner. Furthermore, ATRA significantly reduced the proliferation, invasiveness, tube formation and vascular endothelial growth factor (VEGF) secretion of U87 SLCs. In conclusion, the VM formation ability of SLCs was found to be negatively correlated with differentiation. These results therefore suggested that ATRA may serve as a promising novel agent for the treatment of GBM due to its role in reducing VM formation.

## Introduction

Glioblastoma multiforme (GBM) is one of the most malignant types of primary tumors in the central nervous system, the prognosis of which is poor (1). The growth of GBM is dependent on angiogenesis (2); therefore, antiangiogenic therapy has been considered to be a promising strategy for the inhibition of tumor progression (3). However, there is increasing evidence that traditional antiangiogenic therapy may elicit a greater malignancy in tumors, which was suggested to occur due to vasculogenic mimicry (VM) (4,5). VM is defined as the formation of blood-conducting channels by highly malignant tumor cells (6). A previous study suggested that the presence of VM was a prognostic factor for postoperative survival in GBM patients (7). Consequently, the elucidation of an effective approach for inhibiting VM formation has been the focus of an increasing number of studies (8,9).

The mechanism of VM formation remains to be fully elucidated; however, it has been suggested that cancer stem-like cells (SLCs) may have an important role in this process (10). Cancer SLCs were reported to have the capacity for self-renewal, extensive proliferation, multi-lineage differentiation and tumor initiation (11); these characteristics may also be defined as plasticity. The existence of SLCs in the majority of cancers had been considered to be a possible explanation for the resistance of tumors to traditional radiation and chemotherapy (12,13). Therefore, it was suggested that cancers may be effectively treated through eradicating small subpopulations of SLCs (14).

Differentiation-inducing treatment has been considered to be a promising strategy for the elimination of embryonic cancer SLCs, as it may decrease the ability of these cell to differentiate into multiple linages. A potent differentiation-inducing treatment using all-trans retinoic acid (ATRA) was reported to reduce the tumorigenicity of glioma SLCs, thus exhibiting antitumor effects (15). However, the underlying antitumor mechanism of ATRA remains to be elucidated. It was hypothesized that the antitumor effects of ATRA may be associated with the inhibition of VM formation inhibition. Therefore, the aim of the present study was to investigate the influence of ATRA on the VM formation ability of SLCs derived from the U87 glioblastoma cell line.

## Materials and methods

### Culture of U87

The U87 human glioblastoma cell line (World Health Organization grading guidelines, grade IV) was purchased from the American Type Culture Collection (Manassas, VA, USA) and was maintained in high-glucose Dulbecco’s modified Eagle’s medium (DMEM; Invitrogen Life Technologies, Carlsbad, CA, USA) supplemented with 10% fetal bovine serum (FBS; Invitrogen Life Technologies) in 5% CO_2_ at 37°C. The U87 cell line was used for subsequent experiments up to passage 5.

### U87 SLCs culture and identification

U87 cells were seeded into flasks at a density of 2×10^5^/ml. The serum-free culture medium (stem cell medium) consisted of DMEM/F12 (Invitrogen Life Technologies) supplemented with 20 ng/ml epidermal growth factor (EGF; Sigma-Aldrich, Lyon, France), 20 ng/ml basic fibroblast growth factor (bFGF; Sigma-Aldrich) and B27 (1:50; Invitrogen Life Technologies SAS, Saint Aubin, France). Cultures were incubated in 5% CO_2_ at 37°C and half of the medium was replaced with DMEM every 2 days. When the cultured stem cell spheroids became dark in the center with a diameter of 70–100 *μ*m, they were dissociated enzymatically using 0.25% Trypsin-EDTA (Invitrogen Life Technologies) into single cells and plated at a density of 5×10^3^/cm^2^ in the presence of stem cell medium. Cells were fixed in 4% neutral-buffered formalin (Shanghai Haoran Biotechnology Co., Ltd., Shanghai, China) for 30 min, then blocked with phosphate-buffered saline (PBS)/3% goat serum (Shanghai Haoran Biotechnology Co., Ltd.) for 20 min. Cells were then immunostained overnight at 4°C with the following primary antibodies: Mouse monoclonal immunoglobulin (Ig)G anti-nestin (cat. no. sc-377380; 1:100; Santa Cruz Biotechnology, Inc., Heidelberg, Germany) and rabbit polyclonal IgG anti-CD133 (cat. no. PAB12663; 1:100; Abnova, Taipei, Taiwan). Following washing with DMEM three times, cells were incubated with DyLight™ 594-conjugated goat anti-rabbit IgG (cat. no. 35561; 1:100) or fluorescein isothiocyanate-conjugated goat anti-mouse IgG secondary antibodies (cat. no. A-11001; 1:100; Invitrogen Life Technologies SAS), accordingly, for 1 h at 37°C. Cells were then washed and their nuclei were stained with Hoechst 33342 (Shanghai Haoran Biotechnology Co., Ltd.) for 10 min. Cultures were examined under a Leica DMI4000B fluorescent microscope (Leica Microsystems, Wetzlar, Germany).

### Differentiation assay

U87 SLCs spheres were plated (~8–10 spheres per well) onto sterile a 24-well glass slide coated with poly-L-ornithine (Sigma-Aldrich) in neurosphere medium without EGF and bFGF. The medium was supplemented with 10% FBS (blank control group, BC), FBS combined with 10 nmol/l dimethyl sulfoxide (DMSO; negative control group, NC) or FBS combined with ATRA (dissolved in DMSO; Shanghai Haoran Biotechnology Co., Ltd.) at various concentrations (1, 10 and 100 nmol/l). A non-treated group (NT) of U87 SLCs spheres without any treatment (serum-free) was also established. Spheres were fixed with 4% paraformaldehyde (Shanghai Haoran Biotechnology Co., Ltd.) for 15 min, permeabilized with PBS/0.3% Triton X-100 (Shanghai Haoran Biotechnology Co., Ltd.) and blocked with PBS/3% goat serum for 20 min The spheres were then identified using immunofluorescent staining, as described above, using the following primary antibodies: Chick polyclonal IgG anti-glial fibrillary acidic protein (GFAP; cat. no. ab4674; 1:100; Abcam, Cambridge, MA, USA), rabbit polyclonal IgG anti-β-tubulin III (cat. no. ab18207; 1:100; Abcam) and rabbit polyclonal IgG anti-galactosylceramidase (Galc; cat. no. ab8375; 1:100; EMD Millipore, Billerica, MA, USA). Spheres were then incubated with DyLight™ 488-conjugated goat anti-chick IgG (cat. no. 103-485-155; 1:100; Jackson ImmunoResearch Laboratories, Inc., West Grove, PA, USA) or DyLight™ 594-conjugated goat anti-rabbit IgG (cat. no. 35561; 1:100; Invitrogen Life Technologies SAS) secondary antibodies, accordingly. Following staining with Hoechst 33342, the differentiation rate of U87 SLCs was evaluated by comparing the area of cells stained by Hoechst vs. the area of cells stained by GFAP.

### Proliferation assay

The proliferation ability of cells was evaluated using a cell counting kit (CCK)-8 test (Shanghai Haoran Biotechnology Co., Ltd.). In brief, differentiated cells were plated into single cell suspensions at a density of 1×10^3^ cells/well into wells of a 96-well plate. Each well was then treated with 10 *μ*l CCK-8 every day for 7 days. Cultures were incubated in 5% CO_2_ at 37°C for 4 h following daily CCK-8 administration. Supernatants were obtained by centrifuging at 1,000 × g for 20 min. Subsequently, the absorbance of the supernatant from each well was detected at 450 nm using a microplate reader (model 550; Bio-Rad Laboratories, Inc., Hercules, USA).

### Cell invasion assay

The invasive ability of differentiated cells derived from U87 SLCs spheres was investigated using an invasion assay. The assay was performed using Transwell^®^ cell culture inserts (Invitrogen Life Technologies) according to the manufacturer’s instructions. Cells were then allowed to invade for 24 h. The rate of cell invasion was determined by calculating the mean number of invasive cells from five randomly selected fields of vision for each well (Leica DMI3000B; Leica Microsystems).

### In vitro tube formation assay

A tube formation assay was established, as previously described (16). In brief, wells of a 24-well tissue culture plate were coated with Matrigel^®^ Basement Membrane Matrix (0.1 ml/well; 356234; BD Bioscience, Franklin Lakes, NJ, USA), which was allowed to polymerase at 37°C for 1 h. Differentiated cells were resuspended and seeded onto the Matrigel^®^ at a density of 2.5×10^5^/ml, then incubated without serum in 5% CO_2_ at 37°C for 24 h. Images of the cultures were captured using a Leica inverted microscope (Leica DMI3000B; Leica Microsystems). VM formation ability was determined by counting the total length of tubes per field in five randomly selected fields of vision (magnification, ×100) using Leica Application Suite v3.60 (Leica Microsystems).

### Reverse transcription quantitative polymerase chain reaction (RT-qPCR)

Total RNA was extracted from cells from each group using TRIzol^®^ (Invitrogen Life Technologies SAS) and then verified by electrophoresis using the Sebia Hydrasys 2 agarose gel electrophoresis system (Sebia Inc., Norcross, GA, USA). RNA was then reverse transcribed using the SuperScript™ III First Strand Synthesis System for RT-PCR (Invitrogen Life Technologies). PCR was performed in a 50 *μ*l reaction volume containing 1 *μ*l complementary DNA, 6X reaction buffer, 0.75 mM MgCl_2_, 0.04 mM deoxynucleotide mix, 0.2 pmol/*μ*l forward primer, 0.2 pmol/*μ*l reverse primer and 1 Unit Taq polymerase (Qiagen Multiplex PCR kit, cat. no. 206143; Qiagen, Inc., Valencia, CA, USA) for 35 cycles of 30 sec at 94°C, 58°C and 72°C using a Life ECO Thermal Cycler (BYQ6078; Bioer Technology Co., Ltd., Hangzhou, China). The primers used for amplification were as follows: Vascular endothelial growth factor (VEGF) sense, 5′-CAGCTACTGCCATCCAATC-3′ and antisense, 5′-CAAATGCTTTCTCCGCTCTG-3′ (313 bp); and GAPDH sense, 5′-TGCCAGTGGTAATACGATT-3′ and antisense, 5′-TAGGAATACTGCCATCACAA-3′ (458 bp). GAPDH served as an internal control. RT-qPCR products were electrophoretically analyzed in 1% agarose and visualized with ethidium bromide staining (Shanghai Haoran Biotechnology Co., Ltd.).

### Enzyme-linked immunosorbent assay (ELISA)

VEGF secreted into culture supernatant by treated cells was detected using a HU VEGF ELISA kit (cat. no. KHG0111; Invitrogen Life Technologies). In brief, cells from each group were seeded into 96-well plates at a density of 1×10^4^ cells/well and were cultured for 24 h. Cultures in serum-free medium were incubated in 5% CO_2_ at 37°C for 24 h. The culture medium was then collected and assayed according to the manufacturer’s instructions. Absorbance detection was performed at 450 nm using a microplate reader (model 550; Bio-Rad Laboratories, Inc.).

### Statistical analysis

Values are presented as the mean ± standard deviation of three independent experiments. All statistical analyzes were performed using SPSS 13.0 software (SPSS Inc., Chicago, IL, USA). One-way analysis of variance and least significant difference tests were used to analyze the differences between groups. P<0.05 was considered to indicate a statistically significant difference between values.

## Results

### U87 spheres show properties of SLCs

**Figure 1:**
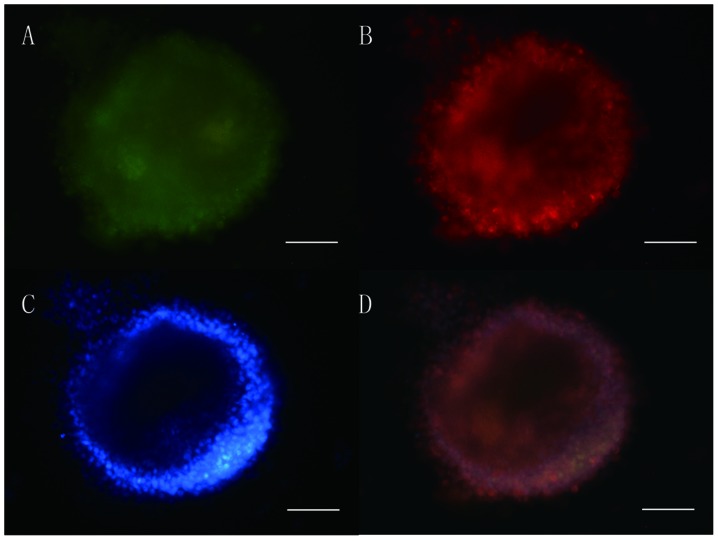
Identification of SLC properties in U87 glioblastoma tumor spheres. Expression of SLC markers (A) CD133 (green) and (B) nestin (red) was determined using immunocytochemistry. (C) Hoechst 33342 (blue) was used to stain the nuclei of U87 cells. (D) Merged image of SLC marker and Hoechst 33342 fluorescence staining (Scale bar, 200 *μ*m). SLC, stem-like cell.

CD133 and nestin were reported to be markers of most tumor SLCs (17). Therefore, in the present study, it was determined whether these markers were expressed in U87 tumor spheres. As shown in Fig. 1, the majority of U87 cells in the spheres were positive for CD133 and nestin.

### ATRA increases the differentiation efficacy of U87 spheres

**Figure 2:**
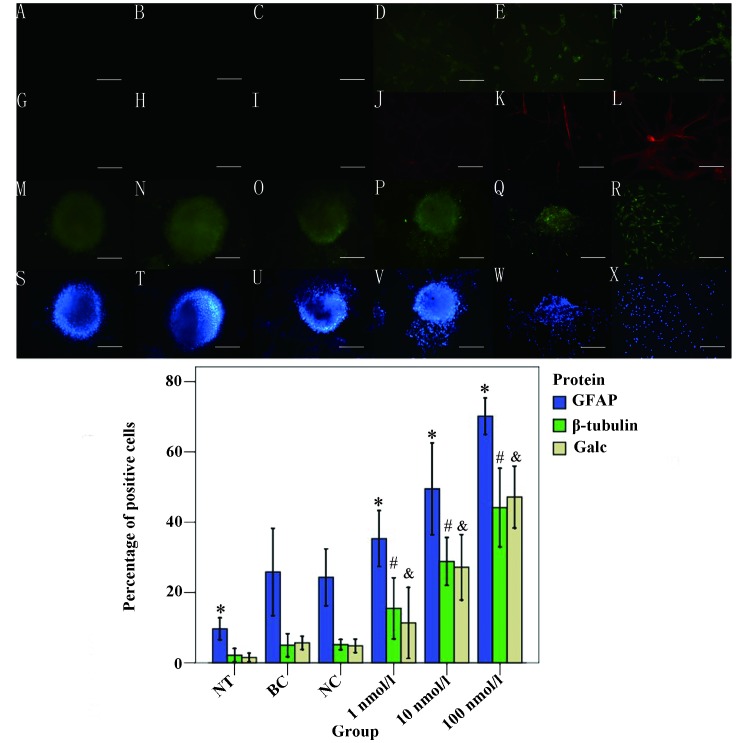
Differentiation of U87 glioblastoma spheres following incubation with different concentrations of ATRA. Immunofluorescence staining was used to determine the expression of (A–F) β-tubulin III, (G–L) Galc and (M–R) GFAP. (S–X) Hoechst 33342 staining of U87 cell nuclei. Spheres were divided into different treatment groups as follows: (A, G, M and S) NT group (serum free); (B, H, N and T) BC group; (C, I, O and U) NC group; (D, J, P and V) 1 nmol/l ATRA group; (E, K, Q and W) 10 nmol/l ATRA group; and (F, L, R and X) 100 nmol/l ATRA group (scale bar, 200 *μ*m). The differentiation rate of spheres was determined by the area of positive-stained cells vs. the total cells stained. ^*^P<0.05 vs. GFAP in BC group, ^#^P<0.05 vs. β-tubulin III in BC group and ^&^P<0.05 vs. Galc in BC group. ATRA, all-trans retinoic acid; Galc, galactosylceramidase; GFAP, glial fibrillary acidic protein; NT, non-treated; BC, blank control; NC, negative control.

In order to determine whether ATRA enhanced the multi-lineage differentiation of U87 spheres, the expression of GFAP, β-tubulin III and Galc was detected using immunofluorescent staining. As shown in Fig. 2, cells in all treatment groups (including the blank and negative control groups) began spreading around, forming small protuberances, connecting with neighboring cells and disrupting the morphology of the spheres; however, these morphological characteristics were not observed in the NT group. By detecting the ratio of GFAP-positive cells to total number of cells, it was demonstrated that 1 nmol/l ATRA (35.33%), 10 nmol/l ATRA (49.50%) and 100 nmol/l ATRA (70.17%) exhibited a significantly more potent ability to promote differentiation compared with that of the BC group (25.83%; P=0.08, P<0.001 and P<0.001, respectively) (Fig. 2). By contrast, the NC group (24.33%) had no significant impact on promoting differentiation compared with that of the BC group (P=0.623). In addition, the NT group (9.67%) had a significantly weaker ability to promote differentiation compared with that of the BC group (P<0.001). The ratio of β-tubulin III-positive cells showed that 1 nmol/l ATRA (15.50%), 10 nmol/l ATRA (28.83%) and 100 nmol/l ATRA (44.17%) had a significantly more potent effect on promoting differentiation compared with that of the BC group (5.00%; all P<0.001) (Fig. 2). By contrast, the NT (2.17%) and NC (5.17%) groups showed no significant impact on promoting differentiation compared with that of the BC group (P=0.218 and 0.940, respectively). Furthermore, the ratio of Galc-positive cells showed that 1 nmol/l ATRA (11.33%), 10 nmol/l ATRA (27.17%) and 100 nmol/l ATRA (47.17%) significantly promoted differentiation in U87 SLCs compared with that of BC group (5.67%; P=0.026, P<0.001 and P<0.001, respectively). By contrast, the NT (1.50%) and NC (4.83%) groups exhibited no significant effect on differentiation compared with that of the BC group (P=0.086 and 0.715, respectively) (Fig. 2).

### ATRA decreases the proliferation of differentiated U87 SLCs

**Figure 3:**
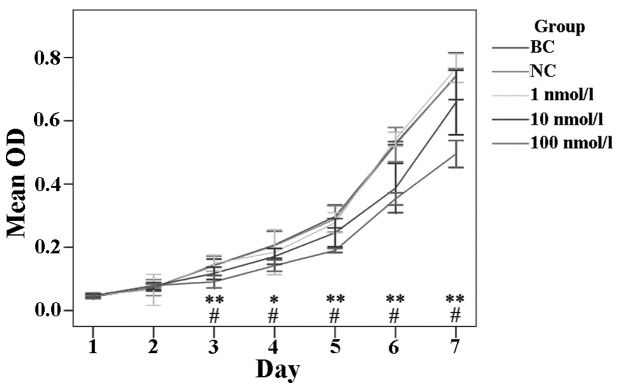
Proliferative ability of differentiated U87 glioblastoma SLCs incubated with different concentrations of ATRA. A Cell Counting Kit-8 assay was used over 7 days to evaluate the proliferation rate of U87 SLCs following treatment with 1, 10 and 100 nmol/l ATRA. ^*^P<0.05 and ^**^P<0.01 10 nmol/l group vs. BC group; and ^#^P<0.01 100 nmol/l group vs. BC group. SLC, stem-like cell; ATRA, all-trans retinoic acid; BC, blank control; NC, negative control; OD, optical density.

A CCK-8 assay was performed in order to determine the influence of ATRA on the proliferation of differentiated U87 SLCs. Proliferation was evaluated by determining the optical density (OD) of each well of ATRA-treated, control and NT cells (Fig. 3). The results showed that cells treated with 10 nmol/l ATRA had a significantly reduced OD compared with that of the BC group on the 3rd day (0.118±0.008; P=0.008), 4th day (0.171±0.010; P=0.030), 5th day (0.246±0.018; P=0.001), 6th day (0.387±0.031; P<0.001) and 7th day (0.658±0.041; P=0.003) of CCK-8 administration. In addition, cells treated with 100 nmol/l ATRA had a significant decreased OD compared with that of the BC group on the 3rd day (0.091±0.008; P<0.001), 4th day (0.142±0.007; P=0.001), 5th day (0.190±0.003; P<0.001), 6th day (0.353±0.008; P<0.001) and 7th day (0.495±0.017; P<0.001). However, the OD of the 10 nmol/l ATRA or 100 nmol/l ATRA groups showed no significant differences on the 1st and 2nd day of the CCK-8 assay compared with that of the BC group. Furthermore, the OD of the NC and 1 nmol/ATRA groups showed no significant difference compared with that of the BC group on any day of the CCK-8 assay (Fig. 3).

### ATRA reduces the invasive and tube formation abilities of U87 SLCs

**Figure 4:**
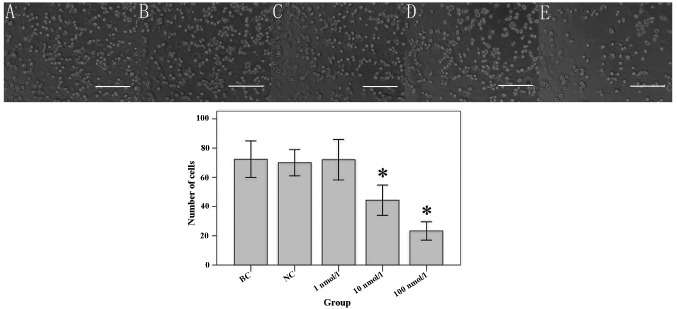
Invasive ability of U87 glioblastoma spheres incubated with different concentrations of ATRA. A cell invasion assay was used to determine the invasive capacity of cells in the (A) BC, (B) NC, (C) 1 nmol/l ATRA, (D) 10 nmol/l ATRA and (E) 100 nmol/l ATRA groups (scale bar, 50 *μ*m). The number of invading cells was then quantified in five fields of vision per group; values are presented as the mean ± standard deviation. ^*^P<0.01 vs. BC group. ATRA, all-trans retinoic acid; BC, blank control; NC, negative control.

**Figure 5:**
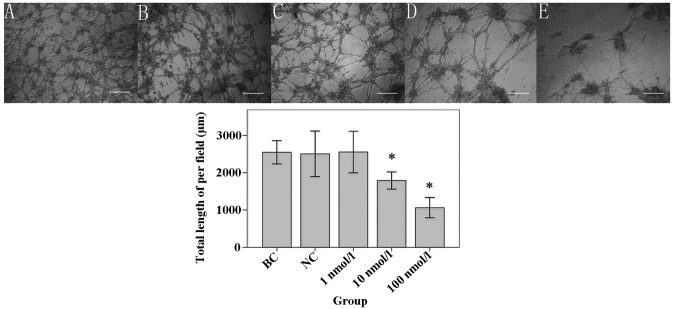
Tube formation ability of U87 glioblastoma spheres incubated with different concentrations of ATRA. A tube formation assay was used to determine the tube formation capacity of cells in the (A) BC, (B) NC, (C) 1 nmol/l ATRA, (D) 10 nmol/l ATRA and (E) 100 nmol/l ATRA groups (scale bar, 500 *μ*m). The total length of vasculogenic mimicry tubes was quantified in five fields of vision per group; values are presented as the mean ± standard deviation. ^*^P<0.01 vs. BC group. ATRA, all-trans retinoic acid; BC, blank control; NC, negative control.

The results of the Transwell^®^ invasion analysis (Fig. 4) showed that the number of invading cells in the 10 nmol/l ATRA group (44.33±4.16) and the 100 nmol/l ATRA group (23.33±2.52) were significantly reduced compared with that of the BC group (72.33±5.03; all P<0.001). However, the number of invading cells in NC group (70.00±3.61) or 1 nmol/l ATRA group (72.00±5.57) was not significantly different compared with that of the BC group (P=0.523 and 0.926, respectively). In addition, the tube formation ability of U87 SLCs was evaluated using a Matrigel^®^ assay (Fig. 5). The results showed that the total length of VM tubes following treatment with 10 nmol/l ATRA (1790.00±93.34 *μ*m) or 100 nmol/l ATRA (1063.33±108.93 *μ*m) was significantly shorter compared with that of the BC group (2547.00±124.85 *μ*m; both P<0.001). By contrast, the total length of the VM tubes in the NC (2505.67±245.98 *μ*m) or 1 nmol/l ATRA (2555.00±224.39 *μ*m) groups were not significantly altered compared with that in BC group (P=0.774 or 0.956, respectively).

### VEGF expression is downregulated in ATRA-treated U87 SLCs

**Figure 6:**
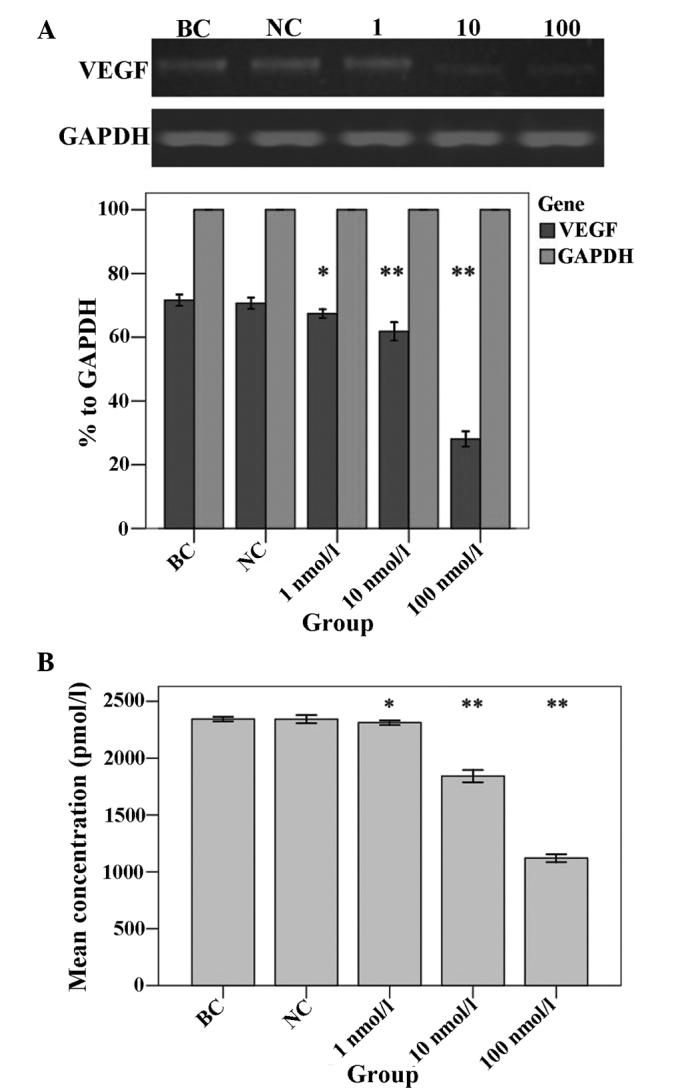
VEGF transcript and protein expression in U87 glioblastoma SLCs incubated with different concentrations of ATRA. (A) Reverse transcription quantitative polymerase chain reaction was used to determine the expression of VEGF transcripts in different treatment groups (BC; NC; and 1, 10 and 100 nmol/l ATRA). GAPDH was used as an internal control. (B) Enzyme-linked immunosorbent assay analysis was used to determine protein expression levels of VEGF in different treatment groups (BC; NC; and 1, 10 and 100 nmol/l ATRA). Values are presented as the mean ± standard deviation. ^*^P<0.05 and ^**^P<0.01 vs. BC group. ATRA, all-trans retinoic acid; VEGF, vascular endothelial growth factor; BC, blank control; NC, negative control.

VEGF expression was detected in U87 SLCs using RT-qPCR (Fig. 6A) and ELISA (Fig. 6B) analysis. The results of the RT-qPCR analysis were normalized to that of GAPDH. These results showed that the expression levels of the VEGF transcript were significantly reduced in groups treated with 1 nmol/l ATRA (67.40%), 10 nmol/l ATRA (61.79%) and 100 nmol/l ATRA (28.04%) compared with those of the BC group (71.61%; P=0.028, P<0.001 and P<0.001, respectively) (Fig 6A). By contrast, the NC group (70.67%) expressed comparable levels of VEGF transcript to those of the BC group (P=0.878). The VEGF protein concentration in the supernatant of the groups treated with 1 nmol/l ATRA (2312.67±7.64 pmol/l), 10 nmol/l ATRA (1841.67±21.55 pmol/l) and 100 nmol/l ATRA (1120.33±14.01 pmol/l) were all significantly reduced compared with that of the BC group (2343.67±7.51 pmol/l; P=0.022, P<0.001 and P<0.001, respectively) (Fig. 6B). By contrast, the NC group (2343.00±14.42 pmol/l) expressed comparable protein levels of VEGF to those of the BC group (P=0.955).

## Discussion

The results of the present study extended the findings of previous studies and verified the hypothesis that ATRA induces the differentiation of GBM cells. Of note, to the best of our knowledge, the present study was the first to report the influence of ATRA on the VM formation ability of U87 glioblastoma cells. In addition, it was demonstrated that ATRA was able to impair the plasticity of U87 SLCs, especially at higher concentration, which was reflected in the increased differentiation as well as the decreased proliferation, invasiveness, tube formation and VM-associated cytokine secretion in U87 SLCs.

Cancer SLCs have been identified in numerous primary culture GBM cells and tumor cell lines originating from GBM (18,19). Tumor spheres which have been derived from cultured cells in serum-free medium in the presence of bFGF and EGF, were found to be rich in cancer SLCs (20). In addition, CD133 and nestin have been confirmed to be molecular markers, which can be used for the identification of SLCs (17). Furthermore, SLCs have been reported to have the multi-lineage differentiation abilities. Xiao *et al* (21) reported that 9L glioma cell spheres derived from mice were able to differentiate into cells positive for GFAP, neuron-specific enolase and Galc, which are representative markers of neuronal, astroglial and oligodendroglial cells; however, in humans, such markers differentiated from SLCs were GFAP, β-tubulin III and Galc (22). In the present study, U87 spheres were found to be positive for CD133 and nestin; in addition, the cultured tumor spheres were able to differentiate into cells positive for GFAP, β-tubulin III and Galc, therefore suggesting the successful induction of U87 SLCs.

Traditional anticancer therapies primarily focused on removing differentiated cancer cells, regardless of SLCs, which are thought to be responsible for tumor progression and relapse (23). In addition, increasing evidence has suggested that cancer SLCs may be incorporated into tumor vascularization, as it has been demonstrated that SLCs were able to give rise to endothelial cells and vascular smooth muscle-like cells (24,25). This therefore suggested that SLCs may function as normal vascular mural cells originated from vascular progenitor cells. Furthermore, El Hallani *et al* (26) reported that SLCs derived from primary GBM were able to form VM-tubes, without the assistance of endothelial cells. Therefore, it is necessary to develop novel strategies targeted at the elimination of SLCs in order to effectively treat cancers and prevent reoccurrences.

A previous study showed that ATRA impaired tumor-induced endothelial-dependent vessel formation *in vitro* and *in vivo* (15). In the present study, the number of U87 cells expressing GFAP, β-tubulin III and Galc was significantly increased following the administration of ATRA, confirming the potent differentiation-inducing ability of ATRA in malignant glioma cells. In addition, differentiated cells derived from U87 SLCs demonstrated significantly depleted tube formation abilities following treatment with ATRA compared with those of the BC group. According to Ma *et al* (27), the VM formation ability of SLCs may also be evaluated using other approaches, such as cell invasiveness. In the present study, cell invasion analysis was also performed, the results of which showed that the invasive ability of U87 SLCs was negatively correlated with the increasing concentrations of ATRA. In addition, a negative correlation was observed between the proliferation of differentiated U87 SLCs and the increasing concentrations of ATRA. This therefore indicated that ATRA significantly impaired the proliferating ability of SLCs in a dose-dependent manner. Overall, it was inferred that ATRA may have an important role in eradicating cancer SLCs, thus may provide an effective therapeutic strategy for the inhibition of endothelial-dependent and endothelial-independent tumor vascularization.

A previous study suggested that SLCs contributed to tumor neovascularization via three possible mechanisms: The production of proangiogenic factors, transdifferentiation and the formation of VM tubes (28). In addition, studies have shown that the expression of certain proangiogenic factors, including VEGF and bFGF, represented the angiogenic ability of tumor cells as well as correlated with the VM formation ability of tumor cells (29,30). It has been reported that downregulation of VEGF disrupted vasculogenic-like networks formed by osteosarcoma cells in three-dimensional culture (31). The results of the present study demonstrated that the expression of VEGF in U87 SLCs was significantly decreased following treatment with ATRA, indicating the anti-VM effect of ATRA. However, there was no significant difference between the results of the 1 nmol/l ATRA group and the BC group in the cell invasion, proliferation and tube formation analyzes, although a significant difference was observed between these two groups in the RT-qPCR and ELISA analyzes. It was therefore suggested that the levels of secreted VEGF may be an indicator of the number of U87 SLCs.

Two major limitations were not addressed in the present study. Firstly, although the U87 cell line has been widely used (32,33), it is still controversial as to whether it is representative of highly malignant GBM. Lee *et al* (34) reported that U87 cells may not represent primary tumors genetically and epigenetically in primary GBM patients, unless the cells were cultured in serum-free medium. Under this consideration, the U87 SLCs used in the present study were cultured in serum-free medium; however, further experiments on primary cell lines are still required. Secondly, convincing *in vivo* studies have not yet been performed. Unlike that found in animals with leukemia, it was reported that ATRA displayed a limited efficacy on the progression of solid tumors (35,36). This may be due to the additional metabolism *in vivo*, which may limit the effectiveness of the administered ATRA dosage in a short period of time and ultimately terminate the effects of ATRA (15). Therefore, further studies are required in order investigate how the ATRA concentration may be maintained *in vivo*. Charoenputtakhun *et al* (37) produced ATRA-loaded lipid nanoparticles for transdermal drug delivery, which obtained promising results. In addition, Hattori *et al* (38) found that ATRA-coated nanoparticles produced an improved curative effect on wound healing compared with that of unpacked ATRA. However, the performance of ATRA-coated nanoparticles requires confirmation in GBM *in vivo*.

In conclusion, the results of the present study demonstrated that ATRA exhibited potent differentiation-promoting effects on U87 SLCs. In addition, ATRA reduced the proliferation and invasiveness of U87 SLCs as well as decreased tube formation and VEGF secretion. Furthermore, the VM formation ability of U87 SLCs was found to be negatively correlated with the differentiation of these cells. These results therefore indicated that ATRA may serve as a promising agent for the treatment of GBM, the mechanism of which proceeds via the inhibition of VM formation.
